# Evaluation of the antinociceptive and anti-inflammatory effects of essential oil of *Nepeta pogonosperma* Jamzad et Assadi in rats

**DOI:** 10.1186/2008-2231-20-48

**Published:** 2012-10-04

**Authors:** Taskina Ali, Mohammad Javan, Ali Sonboli, Saeed Semnanian

**Affiliations:** 1 Department of Physiology, Bangabandhu Sheikh Mujib Medical University, Dhaka, Bangladesh; 2 Department of Physiology, Faculty of Medical Sciences, Tarbiat Modares University, Tehran, Iran; 3 Department of Biology, Medicinal Plants and Drugs Research Institute, Shahid Beheshti University, Tehran, Iran

**Keywords:** Nociception, *Nepeta pogonosperma*, Formalin test, Tail flick test, Essential oil, Inflammation

## Abstract

**Background and the purpose of study:**

Concerning the different effects of essential oils from Nepeta genus on the central nervous system including pain killing effect, this study was designed to evaluate the antinociceptive and anti-inflammatory effects of essential oil of *Nepeta pogonosperma* Jamzad et Assadi *(NP)*, a recently identified species.

**Methods:**

Air-dried aerial parts of *NP* were hydrodistillated and GC-MS analysis of obtained essential oil was conducted. Total 24 male Wister rats weighing 225 ± 25 gm were studied. Essential oil of *NP* was administered intraperitoneally at the doses of 50 mg/kg, 100 mg/kg and 200 mg/kg for the experimental groups. Control rats received equal volume (2 ml/kg) of normal saline. Antinociception was assessed by tail flick test (after 30 minutes) and formalin test (for further 60 minutes). Then the animal was sacrificed and the paw edema was measured using a water plethysmometer.

**Results:**

4aα,7α,7aβ-nepetalactone and 1,8-cineole were found as the main concentrated components of *NP* essential oil. All the doses of *NP* showed antinociception. NP 200 mg/kg reduced the pain sensation in tail flick (p <0.01) and formalin test (p <0.001 in both phases). In paw edema test, NP 100 and 200 mg/kg significantly reduced the inflammation (p <0.01 and p <0.05).

**Conclusion:**

This study reveals that the essential oil of *NP* may minimize both the acute and chronic forms of nociception and may have potent role against inflammation, but the dose should be maintained precisely to obtain the intended effect.

## Introduction

*Nepeta* L. (from Lamiaceae) contains about 300 species, which are distributed in central and southern Europe and in near East, central and southern Asia also. Within them Iran is one of the centers of origin of this genus with 75 species and approximately 53% endemics [1]. The diversity, species richness and variation as well as chemical properties have led to much research into this genus. The extracts of many *Nepeta* species are used in domestic medicine. *N. cataria* L., commonly known as catnip, is the most intensively studied species [2], which is used as a fortifier, a disinfectant and a cure for colds. The extracts of some species are also used because of their diuretic properties and slight bacteriostatic activity, and also in ointments to heal skin disorders of eczema type [3]. Some of the species are widely used in folk medicine because of their expectorant, antiseptic, antitussive, antiasthmatic and febrifuge activities [4-6]. The beverages and infusion prepared from the aerial parts of *Nepeta crispa* Willd. were traditionally used as sedative, relaxant, carminative and also restorative tonic for nervous and respiratory disorders [7]. It has also been shown to have antimicrobial (*N. meyeri*) and anxiolytic (*N. persica*) properties [8].

One of these *Nepeta* plants of Iran is *Nepeta pogonosperma* Jamzad et Assadi, which was identified as new species in 1984 [6]. The essential oil composition of its aerial parts has been reported [9]. The main components of its essential oil were 4aα, 7α, 7aβ-Nepetalactone (57.6%) and 1,8-cineol (26.4%). The dried aerial parts of other species of *Nepeta* were also shown to contain both of these components in different proportion in their essential oil [7,10].

The most important constituent of the essential oil of this *Nepeta* species, 1, 8-cineol, an oxygenated monoterpene, showed inhibitory effect on carrageenan induced paw edema and cotton-pellet induced granuloma in rats [11]. It has also been stated that this terpenoid oxide has strong inhibitory effect on cytokine production in cultured human lymphocytes and monocytes [12]. Moreover, its steroid sparing capacity in bronchial asthma was also determined [13]. A recent report on the essential oil of *Rosmarinus officinalis* L. (a endemic plant in Mexico), which contains 8.58% 1,8-cineole, showed a dose dependent antinociceptive effect in rat model [14]. In addition different isomers of Nepetalactones were reported to have considerable sedative and analgesic activity. 4aα, 7α, 7aβ-Nepetalactone, the key constituent of *Nepeta ceasarea* Boiss, was suggested to have a specific opioid receptor agonistic activity [15]. Again the catnip oil prepared from *Nepeta cataria* showed to have 40% nepetalactone, which was responsible for significant increase in the hexobarbital sleeping time in mice [16].

Different species of Nepeta genus are reported to contain antinociceptive and anti-inflammatory effects. In an study on *Nepeta cataria* essential oil showed of having 79.27% of nepetalactone which might be responsible for its potent antinociceptive and anti-inflammatory activity in mice model [17]. Moreover, extract and fractions from *Nepeta sibthorpil* have been reported to have anti-inflammatory activity in carrageenan induced paw edema model in rat [18]. Recently we reported the analgesic and anti-inflammatory effects of *Nepeta crispa* Willd*.* in animal models [19]. On the basis of these diversified biological activities of the *Nepeta* species and their use in folk medicine, this study was designed to evaluate antinociceptive and anti-inflammatory effects of a newly introduced species*, Nepeta pogonosperma.* We evaluated the antinociceptive effect in both acute and chronic pain models and also investigated its anti-inflammatory activity in an animal paw edema model.

## Materials and methods

### Plant materials

The aerial flowering parts of *Nepeta pogonosperma* Jamzad & Assadi were collected from its wild locality in Qazvin province on 20 May, 2008. The plant was identified by A. Sonboli and a voucher specimen (MPH-1917) was deposited in herbarium of Medicinal Plants and Drugs Research Institute (MPH) of Shahid Beheshti University, Tehran, Iran.

Air-dried aerial parts (100 g) of *Nepeta pogonosperma* were subjected to hydrodistillation using a Clevenger-type apparatus. The essential oil was dried over anhydrous sodium sulfate and stored in sealed vials. The oil was stored at 4°C until the time of analysis and tests. GC-MS analysis of the essential oil was conducted on a Thermoquest-Finnigan Trace GC-MS system equipped with a fused silica DB-1 capillary column (60 m × 0.32 mm i.d., film thickness 0.25 μm). Helium was used as the carrier gas at the constant flow of 1.1 ml/min. The oven temperature was 60°C rising to 250°C at a rate of 5°C/min, then held at 250°C for 10 min; transfer line temperature, 250°C; split ratio was 1/50. The quadrupole mass spectrometer was scanned over the 45–465 amu with an ionizing voltage of 70 eV and an ionization current of 150 μA. The injector and detector (FID) temperatures were kept at 250°C and 280°C, respectively. Retention indices (RI) for all constituents were calculated according to Van den Dool approach, using *n-*alkanes (C_6_ – C_24_) as standards and the essential oil on a DB-1 column under the same chromatographic conditions. The identification of the components was made based on comparison of their mass spectra with those of the internal computer reference mass spectra libraries (Wiley 7.0), as well as by comparison of their retention indices with data published.

### Animals

Twenty four (24) male Wister rats weighing 225 ± 25 g obtained from the Pasteur Institute, Karaj, Iran, were used. They were housed in plexiglass cages as 6 animals per cage with room temperature 24 ± 2°C under a 12 hour light/dark cycle and had free access to water and pellet. The rats were accustomed to the laboratory condition for 4 days before commencement of the experiments. Efforts were made to minimize the number of animals used and their sufferings. All research and animal care procedures were performed according to the international guidelines on the use of laboratory animals and on the basis of codes for ethics in animal research in Tarbiat Modares University

### Drug administration

Animals were treated intraperitoneally (i.p.) with normal saline for the control group or the essential oil of *Nepeta pogonosperma* at the doses of 50, 100 or 200 mg/kg for the experimental groups. The doses were selected based on our previous report on the antinociceptive effects of *N. crispa*[19]. An equal volume of injection of essential oil or normal saline (200 μl/100 g of body weight) was applied for all animals.

### Tail-flick test

Acute antinociceptive effect was assessed based on the method introduced by D’Amour and Smith [20] using a tail-flick apparatus (Harvard Apparatus). The baseline latency was obtained using the mean of similar three consecutive measurements. Normal saline (for control rats) or essential oil (for experimental groups) were injected i.p. immediately after the third pre-drug measurement. Again, test latency was determined after 30 minutes of saline or oil administration (mean of 3 measurements). To minimize tissue damage, a maximum latency of 10 seconds was imposed. Antinociceptive effect was calculated as percent of maximum possible effect (% MPE), as follows [21]:

(1) $$\%\;\text{MPE}=\left[\left(\text{TL}-\text{BL}\right)/\left(\text{CT}-\text{BL}\right)\right]\times 100$$

TL = Test latency; BL = Baseline latency; CT = Cut-off time.

### Formalin test

The formalin test was carried out in a plexiglass observation box, with a mirror placed under the floor (at a 45^o^ angle) to allow a clear view of the paws. Immediately after the recording of the 3rd latency time of the tail flick test, 50 μl of 2% formalin was injected subcutaneously into the plantar aspect of the rat’s right hind paw. The animal was then placed in the observation cage and pain behaviors were recorded for 60 minutes. Nociception was rated using a modification of the original formalin test protocol [22]. Briefly, the pain scoring measurements were as follows: 0 = normal weight bearing on the injected paw; 1 = limping during locomotion or resting the paw lightly on the floor; 2 = elevation of the injected paw; and 3 = licking or biting of the injected paw. The first 5 minutes was considered as early phase and minutes 16 to 60 were considered as the late phase of formalin test [23]. The different behavioral parameters including jerking, flexing and licking were took out from the records as total counts per 5 minutes or total duration in seconds per 5 minutes, respectively.

### Anti-inflammatory test

Anti-inflammatory effects of the essential oil were determined by the formalin-induced paw edema model. The amount of paw edema caused by intra-plantar injection of 2% formalin was used as an indicator of inflammation severity. Following 60 minutes of recording the pain behaviors (about 90 minutes after the i.p administration of the essential oil or saline), the animal was sacrificed. Then the volume of the animals’ right and left hindpaws were measured using a water plethysmometer as mentioned by Fereidoni *et al.* 2001 [24]. Right paw volume was subtracted by left paw volume to obtain the net edema volume.

The rats were not tested more than once and all the experiments were carried out in between 9:00 and 15:00, to minimize the possible influence of circadian changes on rat behavior.

### Statistical analysis

Data obtained for different pain behavior and edema were scrutinized using one way analysis of variance (ANOVA) followed by the Tukey post-hoc test. The results are expressed as mean ± S.E.M and p <0.05 was considered as significant difference of means.

## Results

### Essential oil composition

Although, the main components of *Nepeta pogonosperma* essential oil were reported to be 1,8-cineole and 4aα,7α,7aβ-nepetalactone, concerning the percentage variations in previous reports and the seasonal and local variations in the component of different plants, we analyzed the composition of essential oil applied in this study. Hydrodistilled essential oil of aerial parts of *NP* gave pale yellow oil and forty-one components were identified representing 97.5% of the total oil. Essential oil compounds are presented in Table 1, where compounds are listed in order of their elution on the DB-1 column. The main components of the oil were 4aα,7α,7aα –nepetalactone (14.5%) and 1,8- cineole (31.2%) followed by α-terpineol (5.4%), (E)-α-bisabolene (5.4%), terpinen-4-ol (4.8%), linalool (4.5%) and β-pinene (3.5%).

**Table 1:** **Essential oil composition of *Nepeta pogonosperma***

**Compound**	**RI**	**%**
α-thujene	925	0.3
α-pinene	933	1.4
Sabinene	968	0.7
**β-pinene**	**974**	**3.5**
Myrcene	983	0.7
isobutyl 2-methylbutanoate	993	0.5
isobutyl isovalerate	995	0.9
*p*-cymene	1017	2.7
**1,8-cineole**	**1030**	**31.2**
γ-terpinene	1050	0.4
*trans*-sabinene hydrate	1059	2.1
*cis*-linalool oxide	1064	0.3
**Linalool**	**1089**	**4.5**
2-methylbutyl 2-methylbutanoate	1093	0.7
2-methylbutyl isovalerate	1096	0.3
4-acetyl-1-methyl-1-cyclohexene	1112	0.8
*trans*-pinocarveol	1129	0.8
Pinocarvone	1144	0.8
δ-terpineol	1153	3.1
**terpinen-4-ol**	**1167**	**4.8**
**α-terpineol**	**1179**	**5.4**
Geraniol	1240	0.5
4aβ-7α-7aα-nepetalactone	1325	0.3
**4aα-7α-7aα-nepetalactone**	**1336**	**14.5**
4aα-7α-7aβ-nepetalactone	1346	0.3
4aβ-7α-7aβ-nepetalactone	1361	0.5
geranyl acetate	1364	3.1
β-bourbonene	1383	0.8
trans-caryophyllene	1417	1.1
(E)-β-farnesene	1447	0.7
α-humulene	1450	0.8
germacrene D	1475	0.2
**(*E*)-α-bisabolene**	**1493**	**5.2**
caryophyllene-oxide	1573	2.7
humulene epoxide	1597	0.9
Monoterpene hydrocarbons		9.7
Oxygenated monoterpens		72.2
Sesquiterpene hydrocarbons		8.8
Oxygenated sesquiterpenes		3.6
Others		3.2
Total identified (35 comp.)		**97.5%**

### Tail-flick test

**Figure 1:**
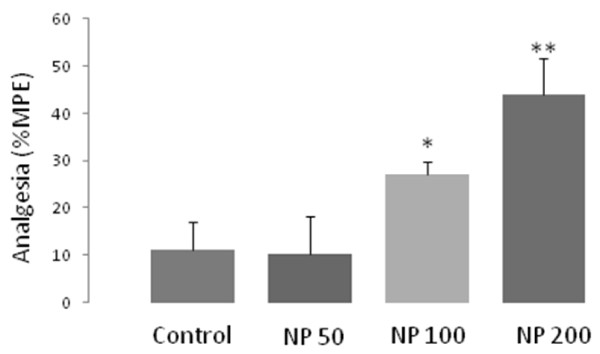
**Antinociceptive effects of different doses (i.p.) of the essential oil of *Nepeta pogonosperma *(NP) on tail flick latency.** Comparison was done on percentage of maximum possible effect (%MPE). Each bar represents the mean ± S.E.M. of 6 rats. * = p <0.05, ** = p <0.01 compared to control.

The effects of 50, 100 and 200 mg/kg doses of *NP* essential oil on acute pain were evaluated using tail flick test. As evaluated at 30 minutes post injection, 50 mg/kg dose of NP did not produce any significant analgesia, but the doses of 100 and 200 mg/kg of NP reduced the acute thermal pain significantly (p <0.01 and p <0.001, respectively). The percentage of maximum possible effect (% MPE) of all the doses were compared to that of the control and presented in Figure 1.

### Formalin test

The effects of systemic i.p. administration of different doses of the essential oil of *NP* on the early and late phases of formalin test were observed. In both phases the pain behaviors were separately analyzed as total jerking frequency, licking duration and flexing duration.

**Figure 2:**
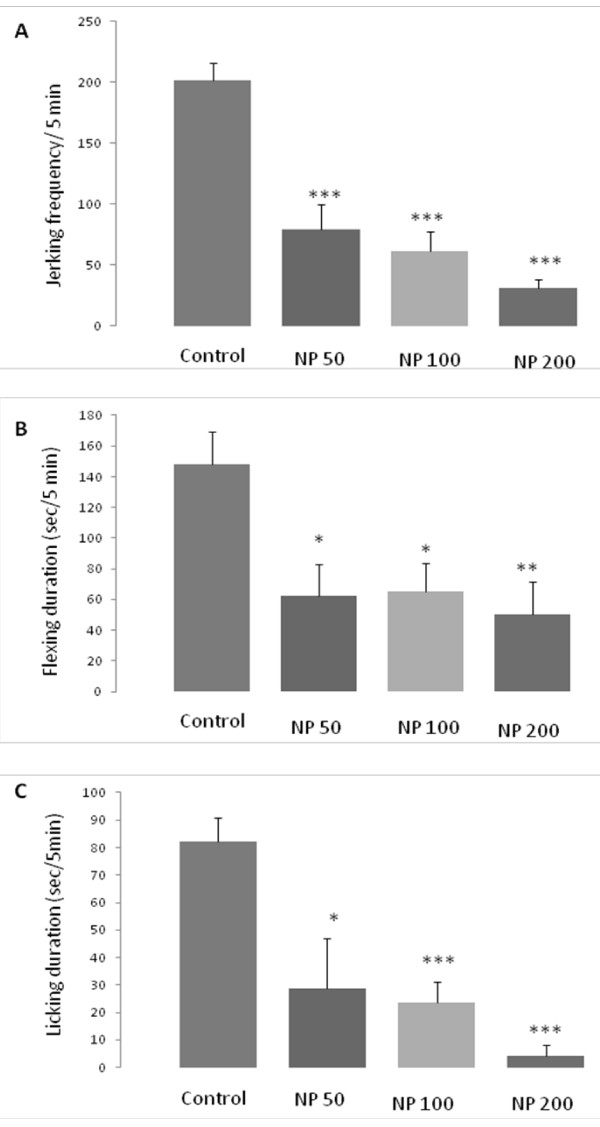
**Antinociceptive effects of different doses of the essential oil of *Nepeta pogonosperma *(NP) in the early phase of formalin test (minutes 0 – 5).** NP doses reduced different pain behaviors including jerking (**A**), flexing (**B**) and licking (**C**). Each bar symbolizes for mean ± S.E.M. for 6 rats. * = p <0.05, ** = p <0.01, *** = p <0.001 compared to control.

As it is mentioned in Figure 2, in the first phase of formalin test 50, 100 and 200 mg/kg doses of NP reduced the jerking frequency (p <0.001, in all doses), flexing duration (p <0.05, p <0.05, p <0.01, respectively) and licking duration (p <0.05, p <0.001, p <0.001, respectively), significantly.

**Figure 3:**
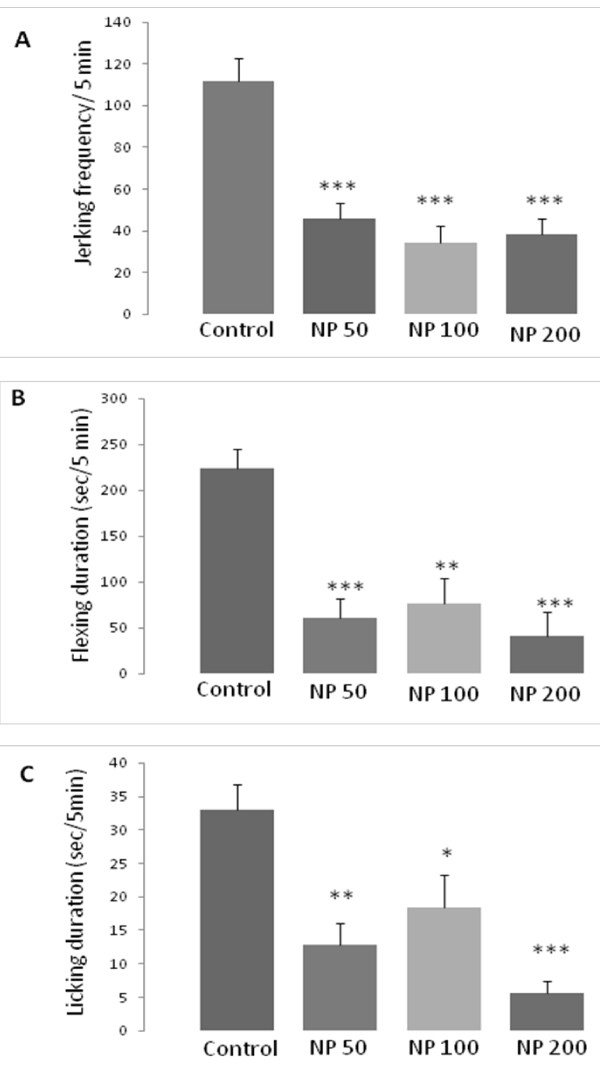
**Antinociceptive effect of different doses of the essential oil of *Nepeta pogonosperma *(NP) in the late phase of formalin test (minutes 16 – 60).** NP doses reduced all pain behaviors including jerking (**A**), flexing (**B**) and licking (**C**). Each bar symbolizes for mean ± S.E.M. for 6 rats. * = p <0.05, ** = p <0.01, ***p <0.001 compared to control.

Again in the late phase of formalin test, as it is mentioned in Figure 3, all the doses of NP reduced the jerking frequency (p <0.001, in all doses), the flexing duration (p <0.001, p <0.01, p <0.001, respectively) and licking duration (p <0.01, p <0.05, p <0.001, respectively), significantly.

### Anti-inflammatory effect

**Figure 4:**
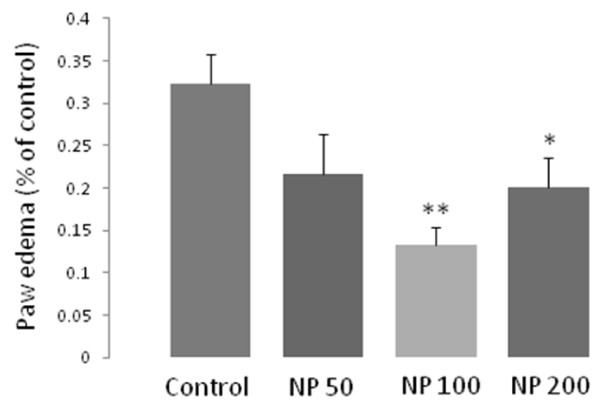
**Anti-inflammatory effects of different doses of the essential oil of *Nepeta pogonosperma *(NP) in formalin-induced paw edema model.** Each bar represents for mean ± S.E.M., n = 6 (experimental) - 9 (control) rats, * = p <0.05, ** = p <0.01, compared to control.

The amount of edema for formalin injected paw was measured at the end of formalin test. As illustrated in Figure 4, all the doses of the oil showed anti-inflammatory effect in the paw edema model though it was significant only in 100 (p <0.01) and 200 (p <0.05) mg/kg of doses of NP.

## Discussion

The management of pain is probably one of the most common and yet most difficult aspects in medical practice. Many improved analgesics and anti-inflammatory agents have been developed, but there is considerable opportunity for conceptual innovation.

We used heat induced and formalin induced pain model for evaluating antinociceptive and formalin induced paw edema model for anti-inflammatory effect of *Nepeta pogonosperma* in experimental rats. Our data demonstrated that the essential oil of this plant elicited potent antinociceptive effects in rats subjected to both the acute thermal (tail-flick) and chronic or persistent formalin pain stimuli and strong anti-inflammatory effect to formalin induced paw edema model.

The Tail flick test is one of the most appropriate techniques to assess the acute somatosensory pain transmission by stimulating thermoreceptors in experimental animal model [25]. King *et al* (1997) showed that this test is sensitive to centrally acting analgesics and supraspinal systems facilitated this tail flicking response which was inhibited by a low dose of morphine [26]. Since our results mentioned potent analgesia in higher two doses of NP in tail flick test, it may be commented that the effective component(s) of this essential oil exerts its antinociceptive effect by modulating the pain transmission in the central nervous system.

Formalin test is one of the appropriate methods for producing and quantifying the chemical pain in the rat model. Pain intensity in this test is dependent on some objective behavioral categories and the observations are converted to numerical values [22]. Subcutaneous injection of formalin induces hindpaw inflammation, which leads to a response characterized by jerking, flexing followed by licking of the affected hindlimb. This characteristic response is considered to be a central nociceptive model, and has been associated with increased levels of chemical mediators in tissue fluids. The level of pain in this model is sensitive to both centrally and peripherally acting analgesics. In the present study, as the i.p. administration of different doses of the essential oil of *NP* inhibited both phases of pain response relative to controls, it may be suggested that it has both the central and peripheral antinociceptive effects. We checked for anti-inflammatory effect of the essential oil and observed reduced formalin induced edema. This finding proved the peripheral antinociceptive effects of the oil bye ameliorating the formalin induced inflammation. Comparing the antinociceptive and anti-inflammatory effects of the lower dose (50 mg/kg), again makes the central analgesic effect plausible. In the other word, the dose which did not produce anti-inflammatory effect exerted significant analgesic effect.

Similar to the previous report [9], NP essential oil contained 1, 8-cineole and 4aα, 7α, 7aβ-Nepetalactone as two major constituent*.* It has been suggested by many investigators that 1, 8-cineole, the most important constituent of *NP* essential oil has potent antinociceptive [14] and anti-inflammatory activity [11,12]. In addition, Aydin and colleagues (1998) suggested that 4aα, 7α, 7aβ-Nepetalactone, might be responsible for the significant analgesic activity and marked sedation in a rat model. They also recommended this nepetalactone might have specific opioid receptor subtype agonist activity [15]. Again in a behavioral study on rats a significant decrease in performance was observed following i.p. administration of nepetalactone enriched fraction [16]. Furthermore the nepetalactone isomers were suggested to be the responsible component of the anti-nociceptive and anti-inflammatory actions of *Nepeta cataria* L. var. citriodora (Becker) Balb. [17]. Moreover, the essential oil of *Nepeta crispa* Willd. which contained 20.3% 4aα, 7α, 7aβ-Nepetalactone and 47.9% 1,8-cineole, showed strong antibacterial, antifungal, antinociceptive and antiinflammatory activity [19,21]. This finding may support the potent anti-inflammatory activity observed in our present experiment. In a previous study, the analgesic activity of the essential oil of *Nepeta italica* L. was showed to be correlated with the amount of 1, 8-cineole [27]. In a recent animal study, cineole has been recommended to reveal an antinociceptive activity comparable to that of morphine in thermal analgesic stimuli [28]. Hence, both of the components of *NP* essential oil, the 4aα, 7α, 7aβ-Nepetalactone and 1, 8-cineole, may be responsible for our experimental findings.

## Conclusion

In conclusion, it may be recommended that the essential oil of *Nepeta pogonosperma* may minimize both the acute and chronic forms of nociception and may have potent role against inflammation, but the dose should be maintained precisely to obtain the intended effect. Although, further experimental study is needed to elucidate the exact component and mechanism responsible for these effects.

## Competing interests

The authors declare that they have no competing interests.

## Authors’ contribution

TA performed the experiments and prepared the draft of manuscript; MJ and SS designed and supervised the study and finalized the MS; AS prepared the plant materials and measured its components. All authors read and approved the final manuscript.

## References

[B1] Jamzad Z Ingrouille M Simmonds M Three new species of Nepeta (Laminaceae) from Iran Taxon 2003 52 92 98

[B2] Grognet J Catnip, its uses and effects, past & present Can Vet J 1990 31 455 456 17423611 PMC1480656

[B3] Javidnia K Miri R Safavi F Azarpira A Shafiee A Composition of the essential oil of *Nepeta persica* Boiss from Iran Flavour Fragr J 2002 17 20 22 10.1002/ffj.1023

[B4] Baser KHC Kirimer N Kerkcuoglu M Demirci B Essential oil of *Nepeta* species in Turkey Chem Nat Comp 2000 36 356 359 10.1023/A:1002832628159

[B5] Newall CA Anderson LA Phillipson JD Herbal Medicines, a Guide for Health Care Professionals 1996 London: Pharmaceutical Press 154

[B6] Jamzad Z Assadi M New species of *Nepeta* and *Ajuga* Ind Jf Botany 1984 2 95 103

[B7] Mozaffarian V A dictionary of Iranian plant names 1996 Tehran: Farhang Moaser

[B8] Joudi L Bibalani GH Exploration of medicinal species of Fabaceae, lamiacea and Asteraceae families in llkhji region, eastern Azerbaijan province (Northwestern Iran) J Med Plant Res 2010 4 1081 1084

[B9] Sefidkon F Akbari-nia A Essential oil composition of Nepeta pogonosperma Jamzad et Assadi from Iran J Essent Oil Res 2003 15 327 328 10.1080/10412905.2003.9698601

[B10] Javidnia K Miri R Safavi F Azarpira A Shafiee A Composition of the essential oil of Nepeta persica Boiss. from Iran Flav Frag J 2002 17 20 22 10.1002/ffj.1023

[B11] Santos FA Rao VS Antiinflammatory and antonociceptive effects of 1,8-cineole a terpenoid oxide present in many plant essential oils Phytother Res 2000 14 240 244 10.1002/1099-1573(200006)14:4<240::AID-PTR573>3.0.CO;2-X 10861965

[B12] Juergens UR Engelen T Racké K Stöber M Gillissen A Vetter H Inhibitory activity of 1,8-cineole (eucalyptol) on cytokine production in cultured human lymphocytes and monocytes Pulm Pharmacol Ther 2004 17 281 287 10.1016/j.pupt.2004.06.002 15477123

[B13] Juergens UR Dethlefsen U Steinkamp G Gillissen A Repges R Vetter H Anti-inflammatory activity of 1,8-cineole (eucalyptol) in bronchial asthma: a double-blind placebo-controlled trial Respir Med 2003 97 250 256 10.1053/rmed.2003.1432 12645832

[B14] Martínez AL González-Truzano ME Pellicer F López-Muñoz FJ Navarrete A Antonociceptive effect and GC/MS analysis of *Roamarinus officinalis* L. essential oil from its aerial parts Planta Med 2009 75 508 511 10.1055/s-0029-1185319 19184968

[B15] Aydin S Beis R OztÜrk Y Baser KH Nepetalactone: a new opioid analgesic from *Nepeta caesarea* Boiss J Pharm Pharmacol 1998 50 813 817 9720633 10.1111/j.2042-7158.1998.tb07145.x

[B16] Harney JW Barofsky IM Behavioral and toxicological studies of cyclopentanoid monoterpenes from *Nepeta cataria* Lloydia 1978 41 367 374 672466

[B17] Ricci EL Toyama DO Lago JHG Romoff P Kirsten TB Reis-Silva TM Bernardi MM Anti-nociceptive and anti-inflammatory actions of *Nepeta cataria* L. var. citridora (Becker) Balb. essenmtial oil in mice J Health Sci Inst 2010 28 289 293

[B18] Miceli N Taviano MF Giuffrida D Trovato A Tzakou O Galati EM Anti-inflammatory activity of extract and fractions from *Nepeta sibthorpil* Bentham J Ethnopharmaco 2004 97 261 266 10.1016/j.jep.2004.11.024 15707763

[B19] Ali T Javan M Sonboli A Semnanian S Antinociceptive and anti-inflammatory activities of essential oil of *Nepeta crispa* Willd. in experimental rat models Nat Prod Res 2012 26 1529 34 10.1080/14786419.2011.565284 21981349

[B20] D’Amour FE Smith DL A method for determining loss of pain sensation J Pharmacol Exo Ther 1941 72 74 79

[B21] Satarian L Javan M Fatollahi Y Epinephrine inhibits analgesic tolerance to intrathecal administrated morphine and increase the expression of calcium-calmodulin-dependent protein kinase II∞ Neurosci Lett 2008 430 213 217 10.1016/j.neulet.2007.10.038 18053645

[B22] Dubisson D Dennis SG The formalin test: a quantitative study of the analgesic effects of morphine, meperidine and brain stem stimulation in rats and cats Pain 1997 4 161 174 10.1016/0304-3959(77)90130-0 564014

[B23] Damaj MI Glassco W Aceto MD Martin BR Antinociceptive and pharmacological effects of metanicotine, a selective nicotinic agonist J Pharmacol Exp Ther 1999 291 390 398 10490929

[B24] Fereidoni M Ahmadiani A Semnanian S Javan M An accurate and simple method for measurement of paw edema J Pharmacol Toxicol Methods 2000 43 11 14 10.1016/S1056-8719(00)00089-7 11091125

[B25] Björkman R Central antinociceptive effects of non-steroidal antiinflammatory drugs and paracetamol. Experimental studies in the rat Acta Anaesthesiol Scand Suppl 1995 103 1 44 7725891

[B26] King TE Joynes RL Grau JW Tail-Flick test: II. The role of supraspinal systems and avoidance learning Behav Neurosci 1997 111 754 767 9267652 10.1037//0735-7044.111.4.754

[B27] Aydin S Demir T Oztürk Y Baser KH Analgesic activity of *Nepeta italica* L Phytother Re 1999 13 20 23 10.1002/(SICI)1099-1573(199902)13:1<20::AID-PTR380>3.0.CO;2-J 10189945

[B28] Liapi C Anifandis G Chinou I Kourounakis AP Theodosopoulos US Galanopoulou P Antinociceptive properties of 1,8-Cineole and beta-pinene, from the essential oil of *Eucalyptus camaldulensis* leaves, in rodents Planta Med 2007 73 1247 1254 10.1055/s-2007-990224 17893834

